# 1-(3-Chloro-2-pyrid­yl)-3-methyl-1*H*-pyrazole-5-carboxylic acid

**DOI:** 10.1107/S1600536809043906

**Published:** 2009-10-28

**Authors:** Hua Cai, Ying Guo, Jian-Gang Li, Yao Wu

**Affiliations:** a College of Science, Civil Aviation University of China, Tianjin 300300, People’s Republic of China

## Abstract

In the title mol­ecule, C_10_H_8_ClN_3_O_2_, the dihedral angle between the pyridine and pyrazole rings is 64.01 (8)°. In the crystal structure, inter­molecular O—H⋯N hydrogen bonds link mol­ecules, forming extended chains along [001]. These chains are, in turn, linked by weak inter­molecular C—H⋯O inter­actions, forming a two-dimensional network perpendicular to the *b* axis.

## Related literature

The title compound was prepared adventitiously as part of our research program related to metal-organic frameworks. See: Lehn (1995 ▶) for background information. For the topologies of metal-organic frameworks, see: Kitakawa *et al.* (2004 ▶); Rosi *et al.* (2005 ▶); Subramanian & Zaworotko (1994 ▶).
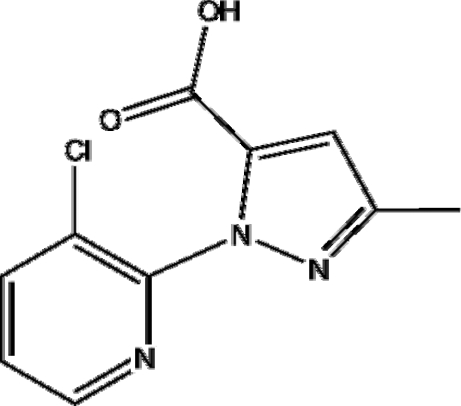


## Experimental

### 

#### Crystal data

C_10_H_8_ClN_3_O_2_
*M* *_r_* = 237.64
Orthorhombic, 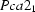

*a* = 8.250 (6) Å
*b* = 11.232 (8) Å
*c* = 11.942 (8) Å
*V* = 1106.6 (13) Å^3^
*Z* = 4
Mo *K*α radiation
μ = 0.33 mm^−1^
*T* = 296 K
0.24 × 0.20 × 0.18 mm

#### Data collection

Bruker SMART APEXII CCD diffractometer
Absorption correction: multi-scan (*SADABS*; Sheldrick, 1996 ▶) *T*_min_ = 0.582, *T*_max_ = 1.000
5084 measured reflections
1943 independent reflections
1754 reflections with *I* > 2σ(*I*)
*R*_int_ = 0.032

#### Refinement

*R*[*F* ^2^ > 2σ(*F* ^2^)] = 0.033
*wR*(*F* ^2^) = 0.075
*S* = 1.04
1943 reflections
147 parameters
1 restraint
H-atom parameters constrained
Δρ_max_ = 0.14 e Å^−3^
Δρ_min_ = −0.13 e Å^−3^
Absolute structure: Flack (1983 ▶) 912 Friedel pairs
Flack parameter: 0.03 (7)

### 

Data collection: *APEX2* (Bruker, 2003 ▶); cell refinement: *SAINT* (Bruker, 2001 ▶); data reduction: *SAINT*; program(s) used to solve structure: *SHELXS97* (Sheldrick, 2008 ▶); program(s) used to refine structure: *SHELXL97* (Sheldrick, 2008 ▶); molecular graphics: *SHELXTL* (Sheldrick, 2008 ▶), *PLATON* (Spek, 2009 ▶) and *DIAMOND* (Brandenburg & Berndt, 1999 ▶); software used to prepare material for publication: *SHELXTL*.

## Supplementary Material

Crystal structure: contains datablocks global, I. DOI: 10.1107/S1600536809043906/lh2933sup1.cif

Structure factors: contains datablocks I. DOI: 10.1107/S1600536809043906/lh2933Isup2.hkl

Additional supplementary materials:  crystallographic information; 3D view; checkCIF report

## Figures and Tables

**Table 1:** Hydrogen-bond geometry (Å, °)

*D*—H⋯*A*	*D*—H	H⋯*A*	*D*⋯*A*	*D*—H⋯*A*
O2—H2⋯N1^i^	0.82	1.93	2.755 (3)	180
C2—H2*A*⋯O1^ii^	0.93	2.36	3.258 (4)	161

Symmetry codes: (i) 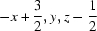; (ii) 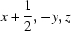.

**Fig. 1.**
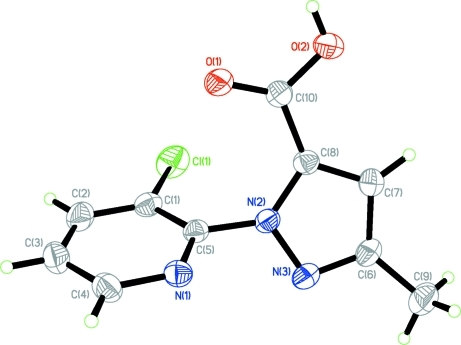
The molecular structure of (I) showing 30% probability ellipsoids.

**Fig. 2.**
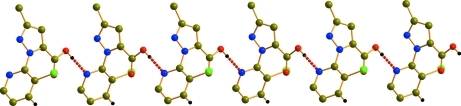
The one-dimensional chain structure of (I), showing O—H···N hydrogen bonds as red dashed lines.

**Fig. 3.**
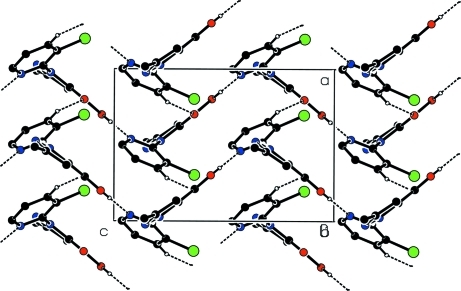
Part of the crystal structure with hydrogen bonds shown as dashed lines.

## supplementary crystallographic information

### Comment

Recently, metal-organic frameworks (MOFs) have attracted great attention (Lehn *et al.*, 1995) because of their intriguing topologies (Subramanian *et al.*, 1994; Kitakawa *et al.*, 2004; Rosi *et al.*, 2005). During our efforts to investigate the assembly of metal-organic coordination frameworks, a new compound, (I), was accidentally generated under hydrothermal conditions and the crystal structure of the title compound (I) is described in this paper. The molecular structure of (I) is shown in Fig. 1. The dihedral angle between the pyridine and pyrazole rings is 64.01 (8)°. The dihedral angle between the mean plane of the pyrazole ring and the plane formed by the atoms C10/O1/O2 is 7.47 (18)°. In the crystal structure, O—H···N hydrogen bonds involving the carboxylic acid O atoms and the 3-chloropyridin-2-yl group N atoms, form one-dimensional chains along [001] (Fig. 2). These chains, are in turn, linked by weak intermolecular C—H···O interactions forming a two-dimensional network perpendicular to the b-axis (Fig. 3).

### Experimental

A mixture of Zn(OAc)_2_.4H_2_O (21.8 mg, 0.1 mmol), 1-(3-chloropyridin-2-yl)-3- methyl-pyrazole-5-carboxylic acid (23.8 mg, 0.1 mmol) in water (10 ml) was heated at 433 K for 3 d in a sealed Teflon-lined stainless steel vessel (20 ml) under autogenous pressure. After the reaction mixture was slowly cooled to room temperature at a rate of 5 K h^-1^, pale-yellow lamellar single crystals suitable for X-ray diffraction were produced.

### Refinement

Although all H atoms were visible in difference Fourier maps, they were placed in calculated positions, with C-H distances in the range 0.93-0.96Å and an O-H distance of 0.82Å, and included in the final refinement in a riding-model approximation, with U_iso_(H) = 1.2U_eq_(C) and U_iso_(H) = 1.5U_eq_(O,C_methyl_)

### Crystal data

**Table:**

C_10_H_8_ClN_3_O_2_	*F*(000) = 488
*M**_r_* = 237.64	*D*_x_ = 1.426 Mg m^−^^3^
Orthorhombic, *P**c**a*2_1_	Mo *K*α radiation, λ = 0.71073 Å
Hall symbol: P 2c -2ac	Cell parameters from 2602 reflections
*a* = 8.250 (6) Å	θ = 3.1–27.5°
*b* = 11.232 (8) Å	µ = 0.33 mm^−^^1^
*c* = 11.942 (8) Å	*T* = 296 K
*V* = 1106.6 (13) Å^3^	Block, colourless
*Z* = 4	0.24 × 0.20 × 0.18 mm

### Data collection

**Table:**

Bruker SMART APEXII CCD diffractometer	1943 independent reflections
Radiation source: fine-focus sealed tube	1754 reflections with *I* > 2σ(*I*)
graphite	*R*_int_ = 0.032
φ and ω scans	θ_max_ = 25.0°, θ_min_ = 1.8°
Absorption correction: multi-scan (*SADABS*; Sheldrick, 1996)	*h* = −9→8
*T*_min_ = 0.582, *T*_max_ = 1.000	*k* = −11→13
5084 measured reflections	*l* = −14→14

### Refinement

**Table:**

Refinement on *F*^2^	Secondary atom site location: difference Fourier map
Least-squares matrix: full	Hydrogen site location: inferred from neighbouring sites
*R*[*F*^2^ > 2σ(*F*^2^)] = 0.033	H-atom parameters constrained
*wR*(*F*^2^) = 0.075	*w* = 1/[σ^2^(*F*_o_^2^) + (0.0254*P*)^2^ + 0.1923*P*] where *P* = (*F*_o_^2^ + 2*F*_c_^2^)/3
*S* = 1.03	(Δ/σ)_max_ < 0.001
1943 reflections	Δρ_max_ = 0.14 e Å^−^^3^
147 parameters	Δρ_min_ = −0.13 e Å^−^^3^
1 restraint	Absolute structure: Flack (1983) 912 Friedel pairs
Primary atom site location: structure-invariant direct methods	Flack parameter: 0.03 (7)

### Special details

**Table:**

Geometry. All e.s.d.'s (except the e.s.d. in the dihedral angle between two l.s. planes) are estimated using the full covariance matrix. The cell e.s.d.'s are taken into account individually in the estimation of e.s.d.'s in distances, angles and torsion angles; correlations between e.s.d.'s in cell parameters are only used when they are defined by crystal symmetry. An approximate (isotropic) treatment of cell e.s.d.'s is used for estimating e.s.d.'s involving l.s. planes.
Refinement. Refinement of *F*^2^ against ALL reflections. The weighted *R*-factor *wR* and goodness of fit *S* are based on *F*^2^, conventional *R*-factors *R* are based on *F*, with *F* set to zero for negative *F*^2^. The threshold expression of *F*^2^ > σ(*F*^2^) is used only for calculating *R*-factors(gt) *etc*. and is not relevant to the choice of reflections for refinement. *R*-factors based on *F*^2^ are statistically about twice as large as those based on *F*, and *R*- factors based on ALL data will be even larger.

### Fractional atomic coordinates and isotropic or equivalent isotropic displacement parameters (Å^2^)

**Table:**

	*x*	*y*	*z*	*U*_iso_*/*U*_eq_	
Cl1	1.19679 (9)	0.18938 (6)	0.13597 (6)	0.0748 (2)	
O1	0.7978 (2)	0.17726 (13)	0.1372 (2)	0.0622 (5)	
O2	0.6983 (2)	0.34151 (14)	0.05851 (17)	0.0664 (5)	
H2	0.6504	0.2936	0.0186	0.100*	
N1	0.9622 (2)	0.18043 (16)	0.42451 (15)	0.0446 (5)	
N2	0.9738 (2)	0.33015 (15)	0.29058 (16)	0.0414 (4)	
N3	1.0231 (2)	0.42331 (17)	0.35467 (17)	0.0490 (5)	
C1	1.1171 (3)	0.1403 (2)	0.26170 (19)	0.0484 (6)	
C2	1.1532 (3)	0.0288 (2)	0.3012 (2)	0.0606 (7)	
H2A	1.2178	−0.0224	0.2594	0.073*	
C3	1.0928 (3)	−0.0067 (2)	0.4034 (3)	0.0625 (7)	
H3	1.1145	−0.0823	0.4314	0.075*	
C4	0.9994 (3)	0.0727 (2)	0.4629 (2)	0.0544 (6)	
H4	0.9607	0.0501	0.5329	0.065*	
C5	1.0179 (2)	0.21361 (19)	0.32469 (18)	0.0396 (5)	
C6	0.9573 (3)	0.5193 (2)	0.3074 (2)	0.0502 (6)	
C7	0.8657 (3)	0.4882 (2)	0.2137 (2)	0.0508 (6)	
H7	0.8092	0.5395	0.1668	0.061*	
C8	0.8758 (3)	0.36675 (19)	0.20469 (19)	0.0421 (5)	
C9	0.9834 (4)	0.6389 (2)	0.3575 (3)	0.0786 (9)	
H9A	1.0755	0.6363	0.4066	0.118*	
H9B	0.8889	0.6617	0.3992	0.118*	
H9C	1.0026	0.6958	0.2991	0.118*	
C10	0.7890 (3)	0.28386 (18)	0.1310 (2)	0.0435 (5)	

### Atomic displacement parameters (Å^2^)

**Table:**

	*U*^11^	*U*^22^	*U*^33^	*U*^12^	*U*^13^	*U*^23^
Cl1	0.0825 (5)	0.0840 (5)	0.0578 (4)	0.0041 (4)	0.0284 (4)	−0.0126 (4)
O1	0.0816 (12)	0.0439 (10)	0.0612 (10)	−0.0104 (8)	−0.0217 (10)	0.0019 (10)
O2	0.0833 (13)	0.0539 (10)	0.0618 (12)	−0.0028 (9)	−0.0294 (11)	0.0010 (9)
N1	0.0460 (11)	0.0480 (11)	0.0399 (11)	−0.0015 (8)	0.0012 (9)	0.0004 (8)
N2	0.0477 (11)	0.0391 (10)	0.0376 (10)	−0.0016 (8)	0.0009 (9)	−0.0041 (8)
N3	0.0497 (11)	0.0477 (11)	0.0496 (11)	−0.0037 (9)	−0.0004 (10)	−0.0108 (9)
C1	0.0471 (13)	0.0541 (14)	0.0441 (13)	0.0011 (11)	0.0002 (10)	−0.0101 (11)
C2	0.0564 (14)	0.0555 (15)	0.0701 (17)	0.0155 (12)	−0.0069 (14)	−0.0158 (13)
C3	0.0659 (17)	0.0482 (13)	0.0734 (18)	0.0074 (14)	−0.0155 (15)	0.0052 (13)
C4	0.0598 (15)	0.0549 (16)	0.0484 (13)	−0.0030 (13)	−0.0059 (11)	0.0096 (11)
C5	0.0368 (11)	0.0429 (12)	0.0391 (12)	−0.0007 (9)	−0.0034 (9)	−0.0053 (9)
C6	0.0511 (13)	0.0416 (13)	0.0578 (15)	−0.0040 (11)	0.0021 (12)	−0.0096 (11)
C7	0.0562 (14)	0.0425 (13)	0.0539 (13)	0.0012 (11)	−0.0029 (12)	0.0017 (11)
C8	0.0451 (12)	0.0438 (13)	0.0373 (11)	−0.0019 (10)	0.0012 (9)	−0.0007 (10)
C9	0.088 (2)	0.0546 (16)	0.093 (2)	0.0004 (16)	−0.0138 (19)	−0.0207 (16)
C10	0.0493 (12)	0.0425 (12)	0.0386 (11)	−0.0034 (10)	0.0030 (11)	0.0035 (12)

### Geometric parameters (Å, °)

**Table:**

Cl1—C1	1.729 (3)	C2—H2A	0.9300
O1—C10	1.202 (2)	C3—C4	1.376 (4)
O2—C10	1.314 (3)	C3—H3	0.9300
O2—H2	0.8200	C4—H4	0.9300
N1—C4	1.330 (3)	C6—C7	1.394 (4)
N1—C5	1.331 (3)	C6—C9	1.486 (4)
N2—N3	1.359 (2)	C7—C8	1.371 (3)
N2—C8	1.369 (3)	C7—H7	0.9300
N2—C5	1.418 (3)	C8—C10	1.468 (3)
N3—C6	1.333 (3)	C9—H9A	0.9600
C1—C2	1.371 (4)	C9—H9B	0.9600
C1—C5	1.383 (3)	C9—H9C	0.9600
C2—C3	1.377 (4)		
			
C10—O2—H2	109.5	C1—C5—N2	123.0 (2)
C4—N1—C5	118.9 (2)	N3—C6—C7	111.0 (2)
N3—N2—C8	111.56 (16)	N3—C6—C9	120.1 (2)
N3—N2—C5	118.17 (18)	C7—C6—C9	128.9 (2)
C8—N2—C5	130.08 (18)	C8—C7—C6	106.2 (2)
C6—N3—N2	105.21 (18)	C8—C7—H7	126.9
C2—C1—C5	119.0 (2)	C6—C7—H7	126.9
C2—C1—Cl1	120.47 (19)	N2—C8—C7	106.01 (19)
C5—C1—Cl1	120.49 (18)	N2—C8—C10	123.1 (2)
C1—C2—C3	119.4 (2)	C7—C8—C10	130.4 (2)
C1—C2—H2A	120.3	C6—C9—H9A	109.5
C3—C2—H2A	120.3	C6—C9—H9B	109.5
C4—C3—C2	118.2 (2)	H9A—C9—H9B	109.5
C4—C3—H3	120.9	C6—C9—H9C	109.5
C2—C3—H3	120.9	H9A—C9—H9C	109.5
N1—C4—C3	122.7 (3)	H9B—C9—H9C	109.5
N1—C4—H4	118.6	O1—C10—O2	124.5 (2)
C3—C4—H4	118.6	O1—C10—C8	124.4 (3)
N1—C5—C1	121.6 (2)	O2—C10—C8	111.10 (19)
N1—C5—N2	115.29 (19)		
			
C8—N2—N3—C6	−0.9 (2)	C8—N2—C5—C1	69.0 (3)
C5—N2—N3—C6	−176.4 (2)	N2—N3—C6—C7	0.2 (3)
C5—C1—C2—C3	−1.3 (3)	N2—N3—C6—C9	178.8 (2)
Cl1—C1—C2—C3	177.8 (2)	N3—C6—C7—C8	0.6 (3)
C1—C2—C3—C4	−0.8 (4)	C9—C6—C7—C8	−177.9 (3)
C5—N1—C4—C3	−0.4 (4)	N3—N2—C8—C7	1.3 (2)
C2—C3—C4—N1	1.7 (4)	C5—N2—C8—C7	176.0 (2)
C4—N1—C5—C1	−1.9 (3)	N3—N2—C8—C10	−172.2 (2)
C4—N1—C5—N2	−179.44 (19)	C5—N2—C8—C10	2.6 (4)
C2—C1—C5—N1	2.8 (3)	C6—C7—C8—N2	−1.1 (2)
Cl1—C1—C5—N1	−176.29 (17)	C6—C7—C8—C10	171.7 (2)
C2—C1—C5—N2	−179.9 (2)	N2—C8—C10—O1	−1.5 (4)
Cl1—C1—C5—N2	1.0 (3)	C7—C8—C10—O1	−173.2 (3)
N3—N2—C5—N1	60.9 (3)	N2—C8—C10—O2	177.4 (2)
C8—N2—C5—N1	−113.5 (3)	C7—C8—C10—O2	5.7 (4)
N3—N2—C5—C1	−116.5 (2)		

### Hydrogen-bond geometry (Å, °)

**Table:**

*D*—H···*A*	*D*—H	H···*A*	*D*···*A*	*D*—H···*A*
O2—H2···N1^i^	0.82	1.93	2.755 (3)	180
C2—H2A···O1^ii^	0.93	2.36	3.258 (4)	161

Symmetry codes: (i) −*x*+3/2, *y*, *z*−1/2; (ii) *x*+1/2, −*y*, *z*.
